# Rapid Breathing as a Pain Obtunder, Etc.

**Published:** 1881-08

**Authors:** W. G. A. Bonwill


### ARTICLE III.

### *Rapid Breathing as a Pain Obtunder in Minor Surgery, Obstetrics, the General Practice of Medicine, and of Dentistry.* (Continued.)

BY W. G. A. BONWILL, D. D. S.

Read before the Philadelphia County Medical Society, May, 1880.

One very interesting case I will state. In extracting seven teeth for a lady who was very *unwilling* to believe my statement as to touch and no pain, I first removed three teeth after having inhaled for one minute, and when fully herself, she stated that she could not understand why there was no pain while she was conscious of each one extracted; it was preposterous to believe such an effect could be possible, as her reason told her that there is connected with tooth extracting, pain in the part, and of severe character, admitting, though, she felt no pain. She allowed one to be removed without anything, and she could easily distinguish the change, and exclaimed, "It is all the difference imaginable!" When the other three were extracted, there was perfect success again as with the first three.

One of the most marked proofs of the effects of rapid breathing was that of a boy of eleven years of age for whom I had to extract the upper and lower first permanent molars on each side. He breathed for nearly a minute, when I removed in about twenty seconds all four of the teeth, without a moment's intermission or stopping the vigorous breathing; and not a murmer, sigh, or tear afterward.

He declared there was no pain, and we needed no such assertion, for there was not the first manifestation from him that he was undergoing such a severe operation.

Another case, the same day, when I had to extract the superior wisdom teeth on both sides for an intelligent young lady of eighteen years, where I had to use two pairs of forceps on each tooth (equivalent to extraction of four teeth,) she was so profoundly affected afterward that she could not tell me what had been done other than that I had touched her four times. She was overcome from its effects for at least a minute afterward. She was delighted.

With such severe tests I fear very little the result in any case where I can have them do as I bid.

There can be no mistake that there is a *specific action* from something. It cannot be personal magnetism or mesmeric influence exerted by me, for such cases are rare, averaging about 10 per cent. only of all classes. Besides, in mesmeric influence the time has much to do with it; whereas, in my cases, it cannot last over a half minute or minute at most. It cannot be fear, as such cases are generally more apt to be hurt the worse. It is not diversion of mind alone, as we have an effect above it.

There is no better way of testing whether pain has been felt than by taking the lacerated or contused gums of the patient between the index finger and thumb and making a gentle pressure to collapse the alveolar borders: invariably they will cry out lustily, *that is pain!* This gives undoubted proof of a specific agent. There is no attempt upon my own part to exert any influence over my patients in any way other than that they shall believe what I say in regard to giving them *no pain* and in the following of my orders. Any one who knows how persons become mesmerized can attest that it is not the *operator who forces them under against their will*, but it is a peculiar state into which any who have within themselves this temperament can place themselves where any one who knows how can have control. It is not the will of the operator. I therefore dismiss this as unworthy of consideration in connection with rapid breathing.

Then you may now ask, To what do I attribute this very singular phenomenon?

Any one who followed, in the earlier part of this paper, the course of the argument in my soliloquy, after twenty years had elapsed from my observation upon myself of the analgesia effects of chloroform, can almost give something of an answer.

That you may the more easily grasp what I shall say, I will ask you, If it be possible for any human being to make one hundred inhalations in a minute and the heart's action is not increased more than ten or twenty pulsations over the normal, what should be the effect upon the brain and nerve centers?

If the function of oxygen in common air is to set free in the blood, either in the capillaries alone, or throughout the whole of the arterial circulation, carbonic acid gas; and that it cannot escape from the system unless it do so in the lungs as it passes in the general current—except a trace that is removed by the skin and kidneys—and that the quantity of carbonic acid gas set free is in exact relation to the amount of oxygen taken into the blood, what effect *must be* manifested where one hundred respirations in one minute are made—five or six times the normal number—while the heart is only propelling the blood a very little faster through the lungs, and *more feebly*—say 90 pulsations at most, when to be in proportion it should be 400 to 100 respirations to sustain life any length of time?

You cannot deny the fact that a definite amount of oxygen can be absorbed and is absorbed as fast as it is carried into the lungs, even if there be one hundred respirations to the minute, while the pulsations of the heart are only ninety! Nature has *made it* possible to breathe so rapidly to meet any emergency; and we can well see its beautiful application in the normal action of both the heart and lungs while one is violently running.

What would result, and that very speedily, were the act of respiration to remain at the standard—say 18 or 20—when the heart is in violent action from this running? Asphyxia would surely end the matter! And why? The excessive exercise of the whole body is setting free from the tissues such an amount of excretivé matter, and carbon more largely than all the others, that, without a relative action of the lungs to admit the air that oxygen may be absorbed, carbonic acid gas cannot be liberated through the lungs as fast as the waste carbon of the overworked tissues is being made by disassimilation from this excess of respiration.

You are already aware how small a quantity of carbonic acid in excess in the air will seriously affect life. Even 2 to 3 per cent. in a short time will prove fatal. In ordinary respiration of 20 to the minute, the average of carbonic acid exhaled is 4-35.

From experiments made long ago by Vierordt—see Carpenter, 524—you will see the relative per cent. of carbonic acid exhaled from a given number of respirations. When he was breathing six times per minute, 5·5 per cent. of the exhaled air was carbonic acid; twelve times, 4-2; twenty-four times, 3·3; forty-eight times,. 3; ninety-six times 2-6.

Remember this is based upon the whole number of respirations in the minute and not each exhalation—which latter could not be measured by the most minute method.

Let us deduct the minimum amount, 2-6 per cent. of carbonic acid when breathing ninety-six times per minute, from the average, at twenty per minute, or the normal standard, which is recorded in Carpenter, p. 524, as 4-35 per minute, and we have retained in the circulation nearly 2 per cent. of carbonic acid; that, at the average, would have passed off through the lungs without any obstruction, and life equalized; but, not having been thrown off as fast as it should have been, it must, of necessity, be left to prey upon the brain and nerve centres; and as 2 to 3 per cent. we are told, will so poison the blood, life is imperilled and that speedily.

It is not necessary we should argue the point as to whether oxygen displaces carbonic acid in the tissues proper or the capillaries. The theory of Lavoisier on this point has been accepted.

We know furthermore, as more positive, that tissues placed in an atmosphere of oxygen will set free carbonic acid, and that carbonic acid has a paralyzing effect upon the human hand held in it for a short time. The direct and speedy effects of this acid upon the delicate nervous element of the brain is so well known that it must be accepted as law. One of the most marked effects is the suspension of locomotion of the legs and arms, and the direct loss of will power, which must supervene before voluntary muscular inactivity, which amounts to partial paralysis in the hands or feet, or peripheral extremities of the same.

Now, that we have sufficient evidence from the authorities that carbonic acid can be retained in the blood by excessive breathing, and enough to seriously affect the brain, and what its effects are when taken directly into the lungs in excess. we can enter upon what I have held as the most reasonable theory of the phenomenon produced by rapid breathing for analgesic purposes; which *theory* was not *first* conceived and the process made to yield to it, but the phenomenon was long observed, and from the repetition of the effects and their close relationship to that of carbonic acid on the economy, with the many experiments performed upon myself, I am convinced that what I shall now state will be found to substantiate my discovery. Should it not be found to coincide with what some may say is physiological truth, it will not invalidate the discovery itself; for of that I am far more positive than Harvey was of the discovery of the circulation of the blood; or Galileo of the spherical shape of the earth. And I ask that it shall not be judged by my theory, but from the practice.

It should have as much chance for investigation as the theory of Jullus Robert Meyer, upon which he founded, or which gave rise to the establishment of one of the most important scientific truths—"the conservation of energy," and finally the "correlation of forces," which theory I am not quite sure was correct, although it was accepted, and as yet I have not seen it questioned.

With all due respect to him I quote it from the sketch of that remarkable man, as given in the *Popular Science Monthly*, as specially bearing on my discovery:

"Mayer observed while living in Java, that the *venous blood* of some of his patients had a singularly bright red color. The observation riveted his attention; he reasoned upon it, and came to the conclusion that the brightness of the color was due to the fact that a less amount of oxidation was sufficient to keep up the temperature of the body in a hot climate than a cold one. The darkness of the venous blood he regarded as the visible sign of the energy of the oxidation."

My observation leads me to the contrary, that the higher the temperature the more rapid the breathing to get clear of the excess of carbon, and hence more oxygenation of the blood which will arterialize the venous blood, unless there is a large amount of carbonized matter from the tissues to be taken up.

Nor must it be denied because of the reasoning as presented to my mind by some outside influence in my soliloquy, when I first exclaimed, "Nature's anæsthetic," where the argument as to the effects of nitrous oxide gas being due to an excess of oxygen was urged, and that common air breathed in excess would do the same thing.

I am not sure that *it* was correct, for the effect of nitrous oxide is, perhaps, due to a deprivation of mechanically mixed air.

Knowing what I do of theory and practice, I can say with assurance that there is not a medical practitioner who would long ponder in any urgent case as to the thousand and one theories of the action of remedies, but would resort to the *practical* experience of others and his own finally. (What surgeon ever stops to ask how narcotics effect their influence?) After nearly thirty years of association with ether and chloroform, who can positively answer as to their *modus operandi?* It is thus with nearly the whole domain of medicine. It is not yet, by far, among the sciences, with immutable laws, such as we have in chemistry. Experimentation is giving us more specific knowledge, and "practice alone has tended to make perfect." Then, gentlemen will not set at naught my assertion and practical results. When I have stated my case in full it is for *you* to disprove both the theory and practice annunciated. So far as I am concerned I am responsible for both.

You will please bear with me for a few minutes in my attempt at theory.

The annulling of pain, and, in some cases, its complete annihilation, can be accomplished in many ways. Narcotics, anæsthetics—local and internal—direct action of cold, and mesmeric or psychological influence, have all their advocates, and each *will surely* do its work. There is one thing about which, I think we can all agree, as to these agencies: unless the *will* is partially and in some cases completely subjugated there can be primary or secondary effect. The voluntary muscles must become wholly or partially paralyzed for the time. Telegraphic communication must be cut off from the brain, that there be no reflex action. It is not necessary there should be separate nerves to convey pleasure and pain any more than there should be two telegraphic wires to convey two messages.

If, then, we are certain of this, it matters little as to whether it was done by corpuscular poisoning and anæmia as from chloroform or hyperæmia from ether.

I think we are now prepared to show clearly the causes which effect the phenomena in "rapid breathing."

The first thing enlisted is the *diversion of the will force* in the act of forced respiration at a moment when the heart and lungs have been in normal reciprocal action (20 respirations to 80 pulsations,) which act could not be made and carried up to 100 respirations per minute without such concentrated effort that ordinary pain could make no impression upon the brain while this abstraction is kept up.

Second. There is a specific effect resulting from enforced respirations of 100 to the minute, due to the *excess of car-*

*bonic acid gas set free from the tissues*, generated by this enforced normal act of throwing into the lungs *five times* the normal amount of oxygen demanded in one minute, when the heart has not been aroused to exalted action, which comes from violent exercise in running or where one is suddenly startled, which excess of carbonic acid cannot escape in the same ratio from the lungs, since the heart does not respond to the proportionate overaction of the lungs.

Third. Hyperæmia is the last in this chain of effects, which is due to the excessive amount of air passing into the lungs preventing but little more than the normal quantity of blood from passing from the heart into the arterial circulation, but damming it up in the brain with its excess of carbonic acid gas to act also directly upon the brain as well as throughout the capillary and venous system, and as well upon the heart, the same as if it were suspended in that gas outside the body.

These are evident to the senses of any liberal observer who can witness a subject rapidly breathing.

Some ask why is not this same thing produced when one has been running rapidly for a few minutes? For a very good reason: in this case the rapid inhalations are preceded by the violent throes of the heart to propel the carbonized blood from the overworked tissues and have them set free at the lungs where the air is rushing in at the normal ratio of four to one. This is not an abnormal action, but is of necessity, or asphyxia would instantly result and the runner drop. Such sometimes occurs where the runner exerts himself too violently at the very outset; and to do so he is compelled to hold his breath for this undue effort, and the heart cannot carry the blood fast enough. In this instance there is an approach to analgesia as from rapid breathing.

Let me take up the first factor—*diversion of will*—and show that nature invariably resorts to a sudden inhalation to prevent severe infliction of pain being felt. It is the panacea to childhood's frequent bruises and cuts, and every one will remember how when a finger has been hurt it is thrust into the mouth and a violent number of efforts at rapid inhalation is effected until ease comes. By others it is subdued by a fit of crying, which if you will but imitate the sobs, will find how frequently the respirations are made.

One is startled, and the heart would seem to jump out of the chest; in quick obedience to nature the person is found making a number of quick inhalations, which subdue the heart and pacify the will by diversion from the cause.

The same thing is observed in the lower animals. I will relate a case:

An elephant had been operated upon for a diseased eye which gave him great pain for which he was unprepared, and he was wrathy at the keeper and surgeon. It soon passed off, and the result of the application was so beneficial to the animal that when brought out in a few days after to have another touch of caustic to the part, he was prepared for it; and just before the touch, he inflated the lungs to their fullest extent, which occupied more time than the effect of the caustic, when he made no effort at resistance and showed no manifestation of having been pained.

In many cases of extraction of the temporary teeth of children, I make them at the instant I grasp the tooth take *one* very violent inhalation, which is sufficient. Mesmeric anæsthesia can well be classified under diversion or subjugation of the will, but can be effected in but a small percentage of the cases. To rely upon this first or primary effect, except in instantaneous cases, would be failure.

The second factor is the one upon which I can rely in such of the cases as come into my care, save when I cannot induce them to make such a number of respirations as is absolutely necessary. The *whole secret of success lies* in the greatest number of respirations that can be effected in from 60 to 90 seconds, and that without any intermission. If the heart, by the *slow method of respiration*, is pulsating in ratio of four to one respiration, *no effect can be induced.*

When the respirations are, say, 100 to the minute, and made with all the energy the patient can muster, and are kept up while the operation is going on, there can hardly be a failure in the minor operations.

It is upon this point many of you may question the facts. Before I tried it for the first time upon my own person, I arrived at the same conclusion from a course of argument, that rapid breathing would control the heart's action and pacify it, and even reduce it below the normal standard, under my urgent respirations.

In view of the many applications made, I feel quite sure in my belief that, inasmuch as the heart's action is but slightly accelerated, though with less force from rapid breathing at the rate of 100 to the minute, there is such an excess of carbonic acid gas set free and crowding upon the heart and capillaries of the brain, without a chance to escape by the lungs, that it is the same to all intents as were carbonic acid breathed through the lungs in common air. Look at the result after this has been kept up for a minute or more. During the next minute the respirations are not more than one or two, and the heart has fallen really below, in some cases, the standard beat, showing most conclusively that once oxygenatian has taken place, and that the free carbonic acid gas has been so completely consumed, there is no involuntary call through the pneumogastric nerve for a supply of oxygen.

If any physiological facts can be proven at all, then I feel quite sure of your verdict upon my side.

There is one thing that goes so far to prove the theory of Lavoisier regarding the action of oxygen in the tissues and capillaries for converting carbon into carbonic acid gas instead of the lungs, as held prior to that time, and still held by many who are not posted in late experiments. At the time I commenced this practice I must confess I knew nothing of it. The study of my cases soon led me to the same theory of Lavoisier, as I could not make the phenomena agree with the old theory of carbonic acid generated only in the lungs.

When Vierordt was performing his experiments upon himself in rapid breathing from six times per minute to ninety-six, I cannot understand why he failed to observe and record what did certainly result—an extreme giddiness with muscular prostration, and numbness of the peripheries of the hands and feet, with suffusion of the face, and such a loss of locomotion as to prevent standing erect without desiring support. Besides, the very great difference he found in the amount of carbonic acid retained in the circulation, the very cause of the phenomena just spoken of.

One thing comes in just here to account for the lack of respiration the minute after the violent effort. The residual air, which in a normal state is largely charged with carbonic acid, has been so completely exhausted that some moments are consumed before there is sufficient again to call upon the will for its discharge.

As to hyperæmia, you will also assent, now that my second factor is explained; but it is so nearly allied to the direct effect of excessive respiration that we can well permit it to pass without argument. If hyperæmia *is present*, we have a more certain and rather more lasting effect.

In conclusion, I will attempt to prognosticate the application of this principle to the cure of many diseases of chronic nature, and especially tuberculosis; where from a diminished amount of air going into the lungs for want of capacity, and particularly for want of energy and inclination to breathe in full or excess, the tissues cannot get clear of their excrementitious material, and particularly the carbon, which must go to the lungs, this voluntary effort can be made frequently during the day to free the tissues and enable them to take nutritions material for their restoration to their standard of health.

Air will be found of far more value than ever before as one of the greatest of factors in nutrition, and which is as necessary as proper food, and without which every organization must become diseased, and no true assimilation can take place without a due amount of oxygen is hourly and daily supplied by this extra aid of volition which has been so long overlooked.

The pure oxygen treatment has certainly performed many cures; yet, when compared to the mechanical mixture and under the direct control of the will, at all times and seasons, there is no danger from excessive oxygenation as where pure oxygen is given. When every patient can be taught to rely upon this great safety valve of nature, there will be less need for medication, and the longevity of our race will be increased with but little dread by mankind of that terrible monster consumption, which seems to have now unbounded control.

When this theory I have here given you to-night is fully comprehended by the medical world and taught the public, together with the kind of foods necessary for every one in their respective occupation, location, and climate, we may expect a vast change in their physical condition and a hope for the future which will brighten as time advances.

The following incident which has but very recently been made known, gives most conclusive evidence of the truth of the theory and practice of rapid breathing.

A Mexican went into the office of a dentist in one of the Mexican cities to have a tooth extracted by nitrous oxide gas.

The dentist was not in, and the assistant was about to permit the patient to leave without removing the tooth, when the wife of the proprietor exclaimed that she had often assisted her husband in giving the gas, and that she would do so in this instance if the assistant would extract the tooth. It was agreed. All being in readiness, the lady turned on, as she supposed, the gas, and the Mexican patient was ordered to breathe as fast as possible to make sure of the full effect and no doubt of the final success. The assistant was about to extract, but the wife insisted on his breathing more rapidly, whereupon the patient was observed to become very dark or purple in the face, which satisfied the lady that the full effect was manifested, and the tooth was extracted, to the great satisfaction of all concerned. While the gas was being taken by the Mexican the gasometer was noticed to rise higher and higher as the patient breathed faster, and not to sink as was usual when the gas had been previously administered. This led to an investigation of the reason of such an anomalous result, when to their utter surprise they found the valve was so turned by the wife that the Mexican had been breathing nothing but common air, and instead of exhaling into the surrounding air, he violently forced it into the gasometer with the nitrous oxide gas, causing it to rise and not sink, which it should have done had the valve been properly turned for the passage of gas into the lungs of the patient.

No more beautiful and positive trial could happen, and might not by accident or inadvertence happen again in a lifetime.

A similar case was related to me, by the dentist in charge, which happened in Arlington, Del., but he could not, as in the case of the Mexican, understand why it was so.—*Scientific American Supplement.*

